# Ocular Manifestations of West Nile Virus

**DOI:** 10.3390/vaccines8040641

**Published:** 2020-11-02

**Authors:** Antoine Rousseau, Oscar Haigh, Imen Ksiaa, Moncef Khairallah, Marc Labetoulle

**Affiliations:** 1 Department of Ophthalmology, Bicêtre Hospital, Public Assistance, Hospitals of Paris, Reference Network for Rare Diseases in Ophthalmology (OPHTARA), 94270 Le Kremlin-Bicêtre, France; marc.labetoulle@aphp.fr; 2 Center for Immunology of Viral, Auto-Immune, Hematological and Bacterial Diseases (IMVA-HB), Infectious Diseases Models for Innovative Therapies (IDMIT), French Alternative Energies and Atomic Commission (CEA), 92260 Fontenay-aux-Roses, France; oscar.haigh@cea.fr; 3 Department of Ophthalmology, Fattouma Bourguiba University Hospital, Faculty of Medicine; Monastir 5019, Tunisia; khay.imen@yahoo.fr (I.K.); moncef.khairallah@yahoo.fr (M.K.); 4 Department of Ophthalmology, University of Monastir, Monastir 5019, Tunisia

**Keywords:** West Nile virus, ocular involvement, chorioretinitis, retinal vasculitis, uveitis

## Abstract

Ocular manifestations are a feature of West Nile virus infection. They mostly occur in association with severe neuroinvasive disease. Linear chorioretinitis is suggestive of the diagnosis and may raise diagnostic suspicion when associated with evocative systemic signs, and in an epidemic context. Various other less specific inflammatory ocular manifestations have been reported, including anterior uveitis, occlusive retinal vasculitis, optic neuritis, and diplopia. The pathophysiology of ocular disease remains unclear, but it reflects the neuroinvasiveness of the disease. Although ocular involvement most often resolves without visual sequelae, some patients may have permanent loss of vision, adding to the need for the development of a specific treatment and/or vaccines.

## 1. Epidemiology and Risk Factors for Ocular Involvement in West Nile Virus Infection

The prevalence of ocular involvement associated with West Nile virus infection (WNVI) is not precisely known. Chorioretinitis is the most frequent ocular manifestation associated with WNVI and occurs in approximately 80% of patients with neuroinvasive disease [1]. The frequency of eye findings in patients with mild or subclinical WNVI remains to be clarified. In a cohort of 111 patients with a history of WNVI, 24% (27/111) had retinal scars compatible with WNV-associated chorioretinitis. Of the 35 patients with an encephalitis presentation, 17 (49%) had retinal lesions, compared to none of the 14 meningitis cases, 9 (25%) of the 36 uncomplicated fever cases, and 1 (4%) of the 26 asymptomatic cases [2].

It is reported that advanced age, immunosuppression, and diabetes are the most important predictors for neuroinvasive disease [3,4]. Khairallah et al. showed that patients over 45 were more at risk for chorioretinitis, and that diabetes was a risk factor for development and severity for both chorioretinitis and occlusive retinal vasculitis [5].

## 2. Pathophysiology

The pathogenesis of WNVI-associated chorioretinitis has been only partially elucidated. As in other viral diseases, it likely results from a combination of direct tissue damage, caused by viral replication, and deleterious effects caused by immune/inflammatory responses to the virus [6,7]. 

WNV may reach ocular tissue either through hematogenous dissemination to the choroidal vasculature, or from a contiguous spread from the central nervous system via the optic nerve fibers to the retina, retinal pigmented epithelium (RPE), and choroid. The latter hypothesis is suggested by the linear distribution of chorioretinal lesions, which follows the anatomical patterns of retinal nerve fibers [8]. 

The eye is an immune-privileged organ that is somewhat protected from systemic infections by the presence of blood–retinal barriers. These peculiar structures might account for the relative rarity of ocular manifestations of WNVI in non-neuroinvasive cases [9].

RPE cells are sensitive to WNVI. Antiviral responses and defense mechanisms in WNV-infected RPE cells have been studied in vitro by some groups with alpha/beta interferons (IFN-alpha/beta), key mediators of the innate immune response against viral infection. Infection of RPE is followed by increased IFN-beta expression and associated with IFN signaling and subsequent inhibition of WNV replication [10]. However, Liu et al. demonstrated that the WNV non-structural NS2A protein was a major inhibitor of IFN-beta, promoter-driven transcription [11,12]. 

Using microarrays and quantitative real-time PCR analysis in WNV-infected RPE cells, Munoz-Erazo et al. identified gene expression involved in immune and antiviral responses, such as chemokine (C-C motif) ligand 2 (CCL2), chemokine (C-C motif) ligand 5 (CCL5), chemokine (C-X-C motif) ligand 10 (CXCL10), and toll like receptor 3 (TLR3). 

Besides, other novel genes regulated by WNVI were identified, including indoleamine 2,3-dioxygenase (IDO1), genes involved in transforming the growth factor-β pathway (bone morphogenetic protein and activin membrane-bound inhibitor homolog (BAMBI)), and genes involved in apoptosis. WNV-infected RPE did not produce any interferon-γ, suggesting that IDO1 is induced by other soluble factors, by the virus alone, or both. Altogether, WNVI of RPE leads to expression of genes that may influence the RPE and therefore outer blood–retinal barrier integrity during ocular infection and inflammation [13]. Consequently, retinal vasculitis may result from immune-mediated mechanisms associated with WNVI and could be facilitated by preexisting retinal vascular lesions, such as diabetic retinopathy [7,14]. 

Although the microglia of the central nervous system plays a role in the pathophysiology of WNV encephalitis [15], the role of retinal microglia in WNV chorioretinitis is yet to be elucidated. 

Ocular involvement usually occurs during the acute phase of the disease, but it can also be a component of congenital infection [16]. 

## 3. Clinical Manifestations

The most frequent ocular manifestation of WNVI is bilateral multifocal chorioretinitis with a typical aspect. However, several ophthalmologic findings have been reported, including anterior uveitis, retinitis, retinal vasculitis, optic neuropathy, and congenital chorioretinal scarring (Table 1) [14,16,17,18,19,20]. 

### 3.1. Chorioretinitis

**Figure 1:**
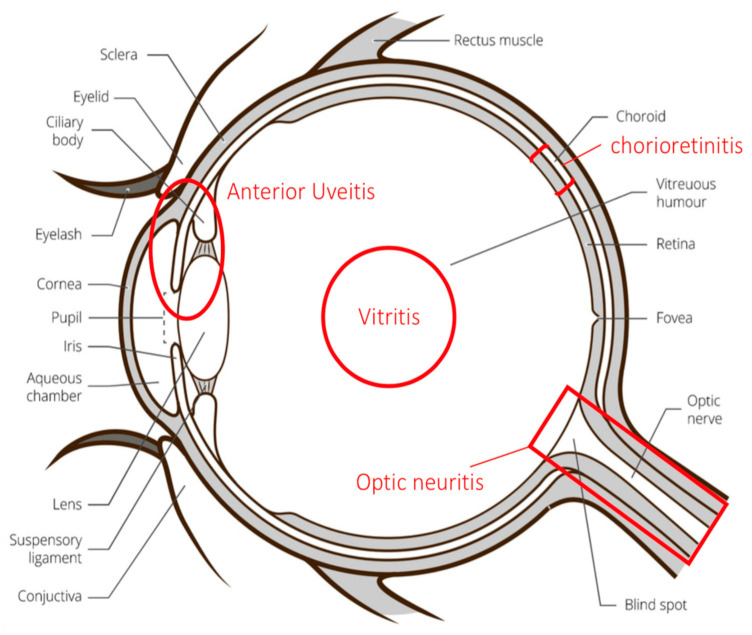
The different ocular structures involved during West Nile virus infection. Anterior uveitis may involve the anterior components of the uvea, which includes the iris and ciliary body.

**Figure 2:**
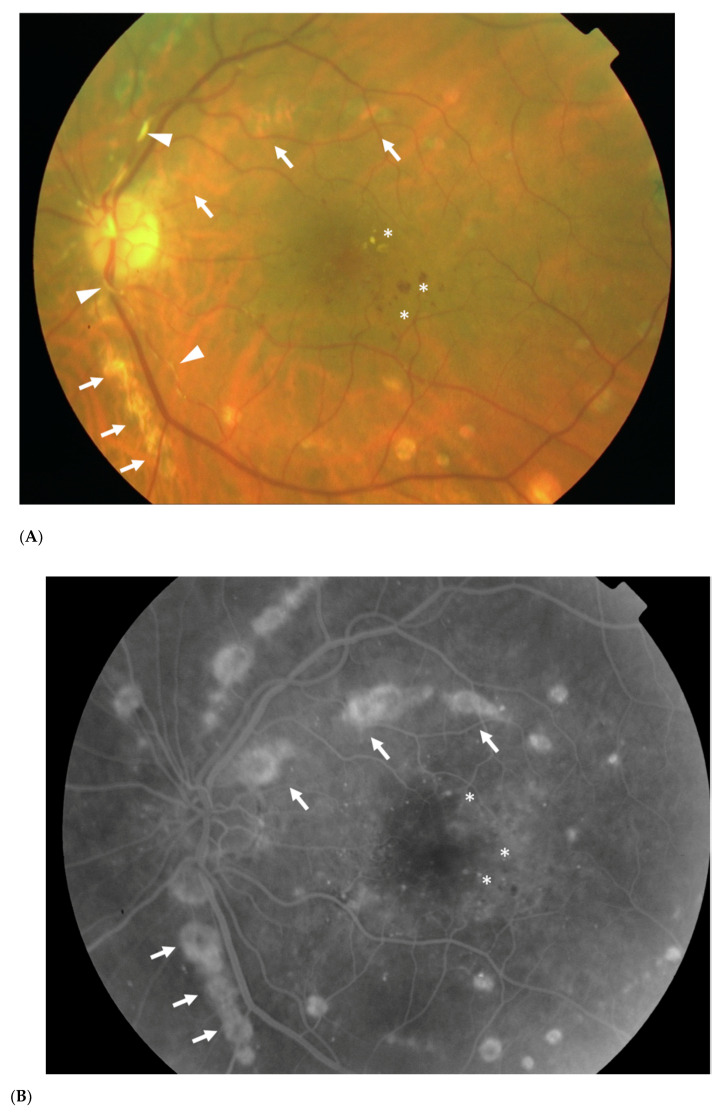

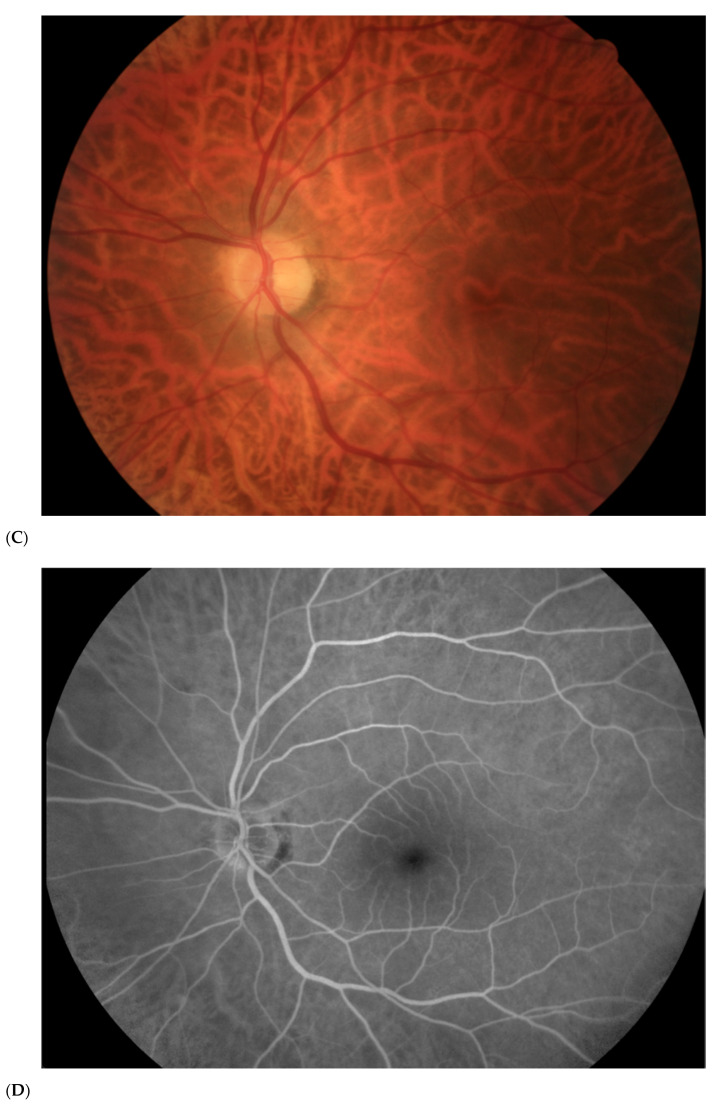
Color fundus photograph (**A**) and late phase fluorescein angiogram (**B**) of the left eye of a 60-year-old diabetic woman with West Nile neuroinvasive disease, showing multifocal chorioretinitis with the typical linear clustering of the chorioretinal lesions (arrows). Fluorescein angiography allows a more precise delineation of the chorioretinal lesions. Note the presence of associated retinal vascular sheathing (arrowheads) and non-proliferative diabetic retinopathy and diabetic maculopathy with macular hemorrhages and hard exudates (asterisks). Fundus photograph (**C**) and fluorescein angiogram (**D**) of an age-matched normal patient are provided for comparison.

Chorioretinitis is defined by an inflammatory condition involving the choroid and the retina (Figure 1). In the context of WNVI, chorioretinitis occurs in almost 80% of patients with neuroinvasive disease [1]. Patients are often asymptomatic. However, they can complain of floaters, and mild to severe vision loss may occur if the posterior pole is involved [19]. Slit lamp examination may disclose accompanying anterior uveitis [21]. On fundoscopic examination, active chorioretinal lesions appear as multifocal, deep, flat, white, or yellowish lesions, with a diameter between 200 and 1000 μm [19]. The lesions are mostly located in the mid-peripheral fundus, although the posterior pole is involved in up to two thirds of eyes. They can be distributed in a non-specific scattered pattern or, more frequently, radiate from the optic nerve in a linear pattern that seems to follow the anatomy of the retinal nerve fiber layer (Figure 2A). This feature is suggestive of the diagnosis, within an epidemic context [8]. The number of lesions can reach up to 50 per eye. An associated mild or moderate vitritis is frequently observed [19,21]. Lesions evolve towards inactive chorioretinal scars, which appear circular and atrophic with or without central pigmentation [19]. 

In the case of chorioretinitis, ophthalmologic evaluation should be completed with multimodal imaging, including fluorescein angiography (FA), indocyanine green angiography (ICGA), fundus autofluorescence, and spectral domain optical coherence topography (SD-OCT). These imaging modalities will provide a better characterization of the lesions and data for further follow-up. In the active stage of the disease, FA shows early hypofluorescence and late staining of the chorioretinal lesions (Figure 2B). Meanwhile, by ICGA, the lesions appear as well-delineated hypofluorescent choroidal spots, which may be more numerous with this technique than those seen clinically or by FA [22]. Fundus autofluorescence imaging shows multiple, well-delineated, mixed hypo- and hyper-autofluorescent, or homogeneously hypoautofluorescent spots. Furthermore, SD-OCT scans of chorioretinal lesions allow analysis of the full chorioretinal thickness, to identify deep retinal involvement with hyper-reflective lesions that may affect the retinal pigment epithelium and outer retinal layers [23]. It also may be used to detect granular hyperreflective specks, located predominantly within the outer and inner nuclear layers that can gradually resolve over time [24]. 

Cicatricial lesions have a peculiar aspect by FA; they take the appearance of a rosette with central hypofluorescence and peripheral hyperfluorescence [17,23], while SD-OCT scans of the scar thickness demonstrate the complete chorioretinal atrophy. 

### 3.2. Retinal Vasculitis

Retinal vascular involvement can occur in association with WNVI and may cause retinal hemorrhages, retinal vascular sheathing (Figure 2A), and in the most severe cases occlusive retinal vasculitis [25,26]. Most reported cases of occlusive retinal vasculitis occurred in elderly patients with a history of diabetes mellitus and were associated with severe irreversible visual loss [25,26]. This severe ocular manifestation can be suspected on clinical examination but should be confirmed using FA. A positive diagnosis involves presence of arterial occlusions, capillary nonperfusion, and neovascularization [14]. SD-OCT is used to demonstrate hyperreflectivity of the inner layers of the retina, corresponding to the ischemic zones [23]. In these patients, OCT angiography, a novel noninvasive imaging technique, allows the detection and precise delineation of areas of retinal capillary nonperfusion in both the superficial and deep capillary plexuses [24].

### 3.3. Other Ocular Manifestations

Kutchey et al. have reported a case of iritis and vitritis without chorioretinitis [27]. Other inflammatory lesions, such as retinitis, macular edema, atrophic lesions of the retinal pigment epithelium, optic disc swelling, optic neuritis, neuroretinitis, and papilledema, have also been reported [14,17,18,19,20,28]. Other neuro-ophthalmic manifestations have been described, including retrogeniculate damage, ocular nerve palsy caused by cranial nerve involvement [14,29,30], and nystagmus secondary to encephalitis [31,32]. Alpert et al. have reported ocular manifestations in the context of congenital infection, where the infant’s mother had developed paraplegia due to WNVI during the second trimester of her pregnancy [16]. While external examination of the child was normal, MRI identified severe damage to the brain, including lissencephaly and a cystic lesion, while fundus exposed peripheral and macular chorioretinal scarring [16].

## 4. Diagnosis and Differential Diagnosis

There are currently no published diagnostic techniques that can be applied specifically to the ocular fluids in WNVI. Serological and molecular biology techniques are detailed elsewhere in this Special Issue. However, the characteristic funduscopic exam and FA findings seen in WNVI-associated chorioretinitis can help establish an early diagnosis of the disease while serologic testing is pending, especially in an epidemic context. 

However, various infectious and inflammatory diseases may also present with chorioretinitis. The most common differential diagnoses include syphilis, tuberculosis, sarcoidosis, histoplasmosis, and idiopathic multifocal chorioretinitis [14,17]. WNV-associated chorioretinitis is usually diagnosed in the context of epidemics, with systemic signs and symptoms (more specifically, neurological involvement). In parallel, the unique linear and multifocal pattern of WNV-associated chorioretinitis is an important clue for diagnosis. 

## 5. Prognosis and Management

Ocular disease associated with WNVI is usually self-limiting, and most patients recover their baseline visual acuity. However, persistent visual loss may occur due to foveal chorioretinal scarring, secondary choroidal neovascularization, vitreous hemorrhage, tractional retinal detachment, macular edema, severe ischemic maculopathy, optic atrophy, or retrogeniculate damage [20,33]. Beardsley and McCanell reported a case of “supposed” viral reactivation in a patient who demonstrated active linear chorioretinal lesions, approximately one year after the initial infection was diagnosed and treated. The patient noted a new onset of visual symptoms, with concomitant elevation of antibody titers to WNV [34]. 

### 5.1. Management

There is, at present, no proven specific treatment or efficient vaccine for WNVI in humans [35]. Specific ophthalmic treatments that may be required include topical steroids and mydriatic agents for anterior uveitis, and peripheral retinal photocoagulation of retinal ischemic zones to prevent neovascularization. Intravitreal injection of anti-vascular endothelial growth factor agents can be used to treat choroidal neovascularization or macular edema [33,36]. Surgical management (pars plana vitrectomy) can be required in case of non-clearing vitreous hemorrhage or tractional retinal detachment [37]. 

### 5.2. Future Directions

Although multiple drug and vaccine candidates have shown promising results in preclinical or early clinical development, there are currently no drugs or vaccines being tested against WNVI in clinical trials. 

Among the drug candidates, the most promising anti-WNV molecules target the conserved enzymatic motifs in viral NS3 protease and NS5 polymerase, and may be effective against different flaviviruses [38]. More recently, ivermectin, an antiparasitic drug, was identified as a potential anti-WNV agent through its binding and inhibition of the host heterodimeric importin (IMP) α/β1 complex, which is normally involved in mediating the nuclear import of key viral and host proteins. In vitro studies have presented anti-WNV activity by this molecule, which remains to be investigated in more robust models [39]. 

Over the last two decades, several vaccine candidates against WNV have been developed [40]. Currently, four veterinary vaccines are licensed for use in horses, and six vaccines have progressed into clinical trials in humans. Among the veterinary vaccines, three are inactivated viruses (WN Innovator™, Zoetis, Parsippany, NJ, USA; Vetera™ WNV Boehringer Ingelheim Vetmedica, Leipzig, Germany; and Prestige^®^WNV, Merck Animal Health, Summit, NJ, USA) and one is a live chimeric virus combining the WNV prM/E gene into a canary pox vector (Recombitek™ Equine WNV, Merial, Athens, GA, USA). Although WNV veterinary vaccines are protective in horses, all require two primary doses and annual boosters to maintain a protective immunity [41,42]. 

To date, human vaccine candidates have not yet been tested beyond Phase II clinical trials. For a human vaccine to be protective in the most vulnerable older age population, it should ideally be strongly immunogenic with a single dose, and without subsequent annual boosters. Of the six human vaccine candidates, the two live attenuated vaccines were unique in their ability to elicit robust immune responses after a single dose [42]. 

## 6. Conclusions

To summarize, WNVI-associated ocular manifestations are features of severe neuroinvasive cases. Linear chorioretinitis is suggestive of a WNVI diagnosis and may raise diagnostic suspicion when associated with evocative systemic signs and in an epidemic context. Although it most often resolves without visual sequelae, some patients may have permanent visual loss, which argues for persistence in the development of a specific treatment/vaccine.

## Figures and Tables

**Table 1:** Ophthalmic manifestations of West Nile virus infection.

Ocular Structure	Clinical Findings
**Anterior segment**	Anterior uveitis
**Posterior segment**	Vitritis Bilateral multifocal chorioretinitis Non occlusive or occlusive retinal vasculitis Retinitis Macular edema Congenital chorioretinal scarring
**Optic nerve** **Other neuro-ophthalmic structures**	Optic neuritis, neuroretinitis, papilledema, optic atrophy Retrogeniculate damage, ocular nerve palsy, nystagmus
